# The seasonality of respiratory syncytial virus in Western Australia prior to implementation of SARS‐CoV‐2 non‐pharmaceutical interventions

**DOI:** 10.1111/irv.13117

**Published:** 2023-03-09

**Authors:** Cara A. Minney‐Smith, David A. Foley, Chisha T. Sikazwe, Avram Levy, David W. Smith

**Affiliations:** ^1^ Department of Microbiology PathWest Laboratory Medicine WA Nedlands Western Australia Australia; ^2^ Wesfarmers Centre of Vaccines and Infectious Diseases, Telethon Kids Institute University of Western Australia Perth Western Australia Australia; ^3^ School of Medicine University of Western Australia Perth Western Australia Australia; ^4^ Infection, and Immunity, Biomedical Sciences University of Western Australia Perth Western Australia Australia

**Keywords:** climate, respiratory syncytial virus, respiratory viruses, seasonality

## Abstract

**Background:**

Respiratory syncytial virus (RSV) seasonality is dependent on the local climate. We assessed the stability of RSV seasonality prior to the SARS‐CoV‐2 pandemic in Western Australia (WA), a state spanning temperate and tropical regions.

**Method:**

RSV laboratory testing data were collected from January 2012 to December 2019. WA was divided into three regions determined by population density and climate: Metropolitan, Northern and Southern. Season threshold was calculated per region at 1.2% annual cases, with onset the first of ≥2 weeks above this threshold and offset as the last week before ≥2 weeks below.

**Results:**

The detection rate of RSV in WA was 6.3/10,000. The Northern region had the highest detection rate (15/10,000), more than 2.5 times the Metropolitan region (detection rate ratio 2.7; 95% CI, 2.6–2.9). Test percentage positive was similar in the Metropolitan (8.6%) and Southern (8.7%) regions, with the lowest in the Northern region (8.1%). RSV seasons in the Metropolitan and Southern regions occurred annually, with a single peak and had consistent timing and intensity. The Northern tropical region did not experience a distinct season. Proportion of RSV A to RSV B in the Northern region differed from the Metropolitan region in 5 of the 8 years studied.

**Conclusions:**

Detection rate of RSV in WA is high, especially in the Northern region, where climate, an expanded at‐risk population and increased testing may have contributed to greater numbers. Before the SARS‐CoV‐2 pandemic, RSV seasonality in WA was consistent in timing and intensity for the Metropolitan and Southern regions.

## 1 BACKGROUND

Respiratory syncytial virus (RSV) is a leading cause of respiratory illness in all ages. RSV is a significant source of childhood morbidity, with an estimated 3 million children under 5 hospitalised annually.^1^ Mortality is relatively uncommon in children and is predominantly restricted to low‐resource settings.^1^ In contrast, mortality in hospitalised older adults is high, with RSV lower respiratory tract infection reaching more than 5%.^2^ RSV can be classified into two groups, RSV‐A and RSV‐B, with the majority group varying between seasons.

RSV seasonality can vary between regions, driven by the prevailing climate.^3^ In areas with a temperate climate, RSV epidemics typically occur annually, peaking in the winter months. The associated cold and dry conditions enhance transmission by increasing viral stability on surfaces.^3^ Areas with tropical climates have more stable year‐round activity, with increases observed in the warm and high humidity rainy season, mediated by enhanced contact‐mediated transmission.^3^, ^4^, ^5^

Western Australia (WA), the largest State in Australia, covers a geographical area of approximately 2.6 million square kilometres, spanning temperate and tropical regions. The lower part of the State, comprising the Metropolitan area and Southern regions, experiences a temperate climate, with the coolest months occurring from June to August during the southern hemisphere winter. The northern part of the State experiences a tropical climate, with the rainy season occurring from November to April. Travel between metropolitan areas and regions is frequent, facilitating constant population mixing between regions.

In 2020, SARS‐CoV‐2‐related non‐pharmaceutical interventions were associated with an absent winter RSV season in WA and a subsequent out‐of‐season surge.^6^, ^7^ An improved understanding of RSV seasonality in the region is required to contextualise the significance of this shift. In this report, we assessed the stability of RSV seasonality in WA, by region and viral subtype, between 2012 and 2019.

## 2 METHODS

Laboratory data were collected for all RSV tests from January 2012 to December 2019 from PathWest Laboratory Medicine, the state reference laboratory. This laboratory provides respiratory virus testing for all WA public hospitals as well as some community‐based healthcare in regional areas.^8^ All clinical samples arriving during the study period requesting respiratory virus testing were tested by RT‐PCR using either an in‐house assay^9^ or GeneXpert Xpress Flu/RSV (Cepheid, USA) for RSV, influenza, human metapneumovirus and parainfluenza viruses 1–3. Until June 2018, patients attending the state tertiary paediatric hospital were also tested by direct immunofluorescence (IMAGEN, ThermoFisher Scientific, Australia) in addition to RT‐PCR. A portion of RSV‐positive samples had an RSV subtype determined by subtype‐specific RT‐PCR.^9^ Individuals with multiple positive and negative tests were counted once per calendar month.

### 2.1 Geographical groupings

**FIGURE 1:**
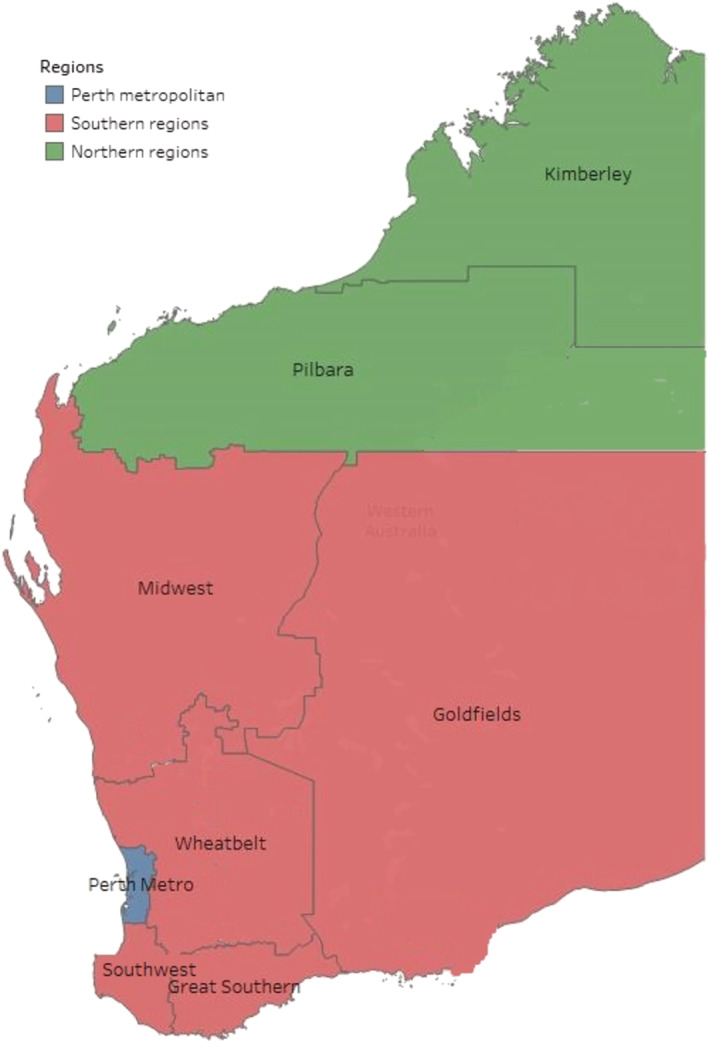
Western Australia by region. Kimberley and Pilbara comprise the Northern region, with Midwest, Goldfields, Wheatbelt, Southwest and Great Southern comprise the Southern region.

The residential postcode at the time of sample collection was used to determine the geographical location. Patients with no address listed and out‐of‐state postcodes were excluded from this analysis. The state was divided into three geographical areas, determined by population density and climate (Figure 1): Metropolitan region, Northern region and Southern region. Based on 2016 census data, the Metropolitan region has a population of 1.9 million, accounting for 80% of WA's total.^10^, ^11^ The Northern region, situated north of the Tropic of Capricorn, has a population of approximately 94,000.^12^ The Southern regions, situated mostly below the Tropic of Capricorn, experience a temperate climate, with cooler weather occurring June–August and has a total population of approximately 430,000.^13^, ^14^, ^15^

### 2.2 Analysis

Season onset and offset threshold was set at 1.2% of the annual total RSV cases for each region and year.^16^ Season onset was defined as the first of two consecutive weeks where RSV cases exceeded this threshold. Season offset was defined as the last week above this threshold before declining below this level for more than two consecutive weeks. The intensity was defined as the highest number of cases per week per year. In instances with more than 1 week with equal peak cases, the median of those weeks was used for timing. Statistical comparisons between regions were calculated using the chi‐square test. Grubbs test was used to detect outliers in season intensity within regions, as measured by the number of cases in the peak week. Detection rate was calculated using population data as the denominator,^10^, ^11^, ^12^, ^13^, ^14^, ^15^ and detection rate ratios were used to compare detections between regions. A *p*‐value of less than 0.05 was considered significant in all statistical tests.

## 3 RESULTS

Between 2012 and 2019, PathWest tested 144,590 WA individuals for RSV, 12,424 (8.6%) of which were positive (Table 1; Table S1). Age of cases were predominantly children, with 41.5% under 12 months, 28.3% 1–4 years, 19% 19.3 years and 10.9% over 65 years; 52.1% of cases were male. Most cases (68.5%) were detected in the more populous metropolitan region. The mean detection rate of RSV during the study period in WA was 6.3 per 10,000. The metropolitan region's mean annual cases per 10,000 population was 5.5 (range, 4.3 to 7.2). The mean cases per 10,000 was 8.1 (range, 6.4 to 12.4) in the Southern Region. The highest rates were observed in the Northern region, with a mean of 15 per 10,000 (range, 11.2 to 20.7). Rates were consistently higher each year in the Northern regions and lower in the Metropolitan region, contributing to a detection rate ratio (DRR) of 2.7 (95% confidence interval [CI], 2.6–2.9). Overall, the proportion positive was similar between the Metropolitan region (8.6%) and the Southern region (8.7%). The lowest percentage‐positivity was observed in the Northern region (8.1%).

**TABLE 1:** RSV test percentage positivity and rates by region per year.

	All		Metropolitan		Southern			Northern		
Year	% pos (n)	Per 10,000	% pos (n)	Per 10,000	% pos (n)	Per 10,000	Metropolitan DRR (95% CI)	% pos (n)	Per 10,000	Metropolitan DRR (95% CI)
2012	9.4 (1520)	6.2	8.9 (1032)	5.3	11.3* (345)	8	DRR 1.5 (1.3–1.7)	9.8 (143)	15.2	DRR 2.9 (2.4–3.4)
2013	9.2 (1409)	5.7	9 (1029)	5.3	9.9 (275)	6.4	DRR 1.2 (1.1–1.4)	9.5 (105)	11.2	DRR 2.1 (1.7–2.6)
2014	7.9 (1526)	6.2	8.1 (1093)	5.6	7.7 (292)	6.8	DRR 1.2 (1.1–1.4)	7.6 (141)	15	DRR 2.7 (2.2–3.2)
2015	8.9 (1895)	7.7	9.1 (1404)	7.2	8.1 (348)	8.1	DRR 1.2 (1–1.3)	9 (143)	15.2	DRR 2.1 (1.8–2.5)
2016	8.1 (1660)	6.7	8.1 (1177)	6.1	8.1 (326)	7.6	DRR 1.3 (1.1–1.4)	8.2 (157)	16.7	DRR 2.8 (2.3–3.3)
2017	8.7 (1271)	5.2	9.1 (830)	4.3	8.8 (322)	7.5	DRR 1.8 (1.5–2)	6.7** (119)	12.7	DRR 3 (2.4–3.6)
2018	9.3 (1335)	5.3	9.7 (869)	4.5	8.7 (341)	7.9	DRR 1.8 (1.6–2)	8.1 (125)	13.3	DRR 3 (2.4–3.6)
2019	7.8 (1808)	7.3	7.7 (1081)	5.6	8.3 (532)	12.4	DRR 2.2 (2–2.5)	7 (195)	20.7	DRR 3.7 (3.2–4.4)
Mean	8.6 (1553)	6.3	8.6 (1064)	5.5	8.7 (348)	8.1	DRR 1.5 (1.4–1.5)	8.1 (141)	15	DRR 2.7 (2.6–2.9)

*Note*: Rate calculated per 10,000 resident population. *N* = number RSV detected per year. Case and population numbers are supplied in Table S1.

Abbreviations: %pos, percentage test positivity; 95% CI, 95% confidence interval; Metropolitan DRR, detection rate ratio compared with Metropolitan region.

* Proportion statistically higher than the two other regions (*p* < 0.05).

** Proportion statistically lower than the other two regions (*p* < 0.05).

### 3.1 Seasonality

**FIGURE 2:**
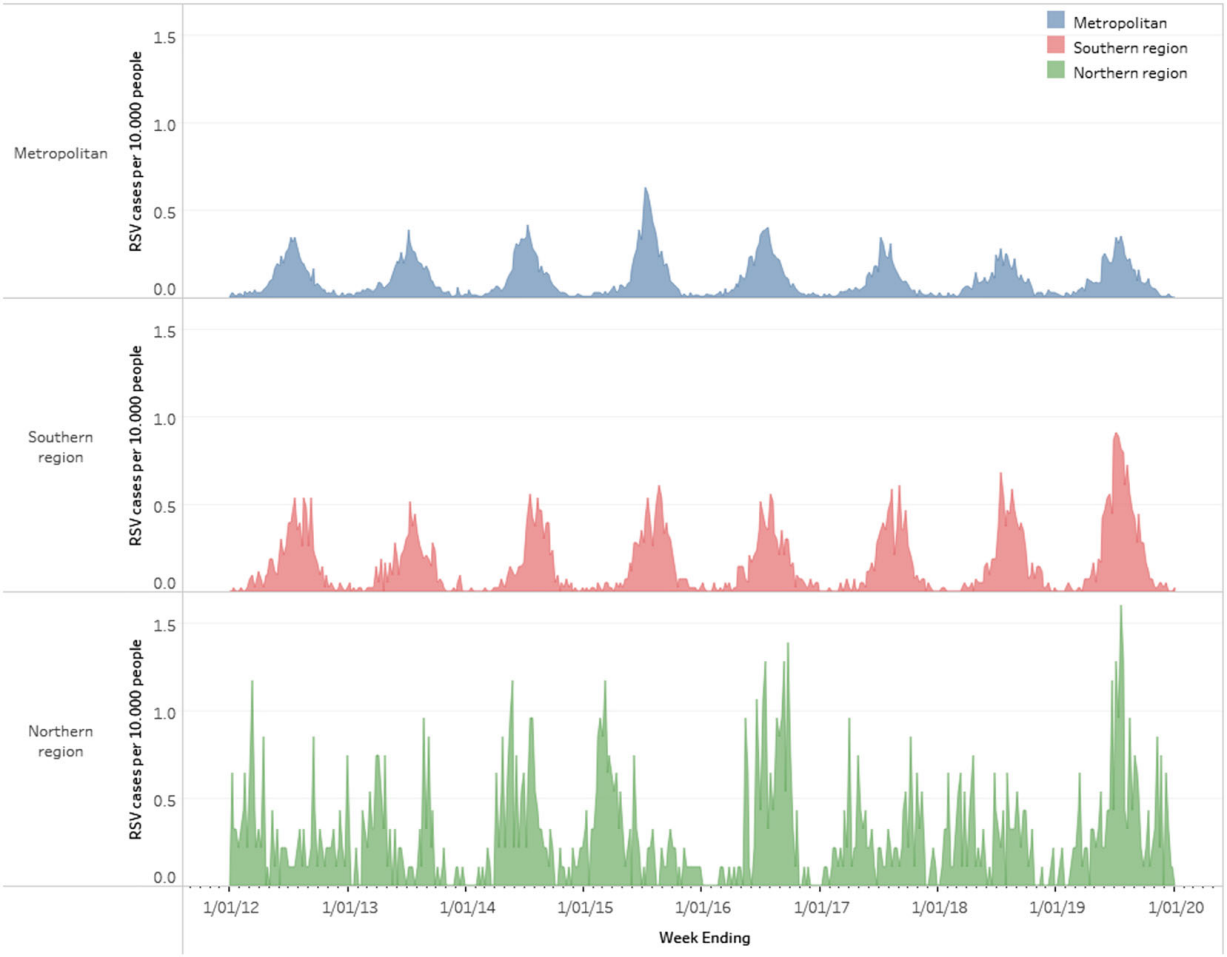
Weekly RSV cases per 10,000 people in WA by geographical area. Census data for 2016 were used to determine rates.

**FIGURE 3:**
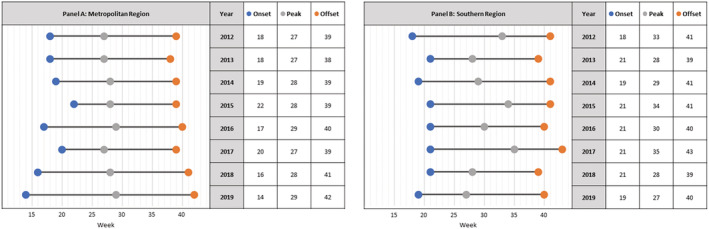
Onset, peak and offset weeks for Metropolitan (A) and Southern regions (B).

The Metropolitan region experienced a single annual peak each year, occurring in the winter months (Figure 2). Season onset, peak and offset were stable across years (Figure 3), with season onset in weeks 14–22 (mean = 18), peaking in weeks 27–29 (mean = 28) and declining by weeks 38–42 (mean = 40).

The Southern region also experienced a single annual peak. Season onset ranged from week's 18–21 (mean = 20), peaking in weeks 28–35 (mean = 31) and season offset in weeks 39–43 (mean = 41). Season onset for the Southern region occurred after the Metropolitan region in five of the eight seasons studied (range 3–5 weeks later). The peak for the Southern Regions followed that of the Metropolitan area in six of the eight seasons (range 1–8 weeks later). Season offset occurred first in the Metropolitan region for five out of eight seasons (range 1–4 weeks earlier).

There was a minimal variation of intensity for the Metropolitan and Southern regions for seven of the eight seasons examined. The peak weekly cases for the Metropolitan region varied from 55–82 cases for all years except 2015, which was an outlier with 125 cases. The peak weekly cases in the Southern region varied from 22 to 29, with an outlier with 39 cases in 2019.

Season onset and offset were not defined for the Northern region, as data analysis could not determine a seasonal pattern. The northern regions did not experience an annual peak but instead had continued activity throughout the year (Figure 2).

### 3.2 RSV subtypes

**FIGURE 4:**
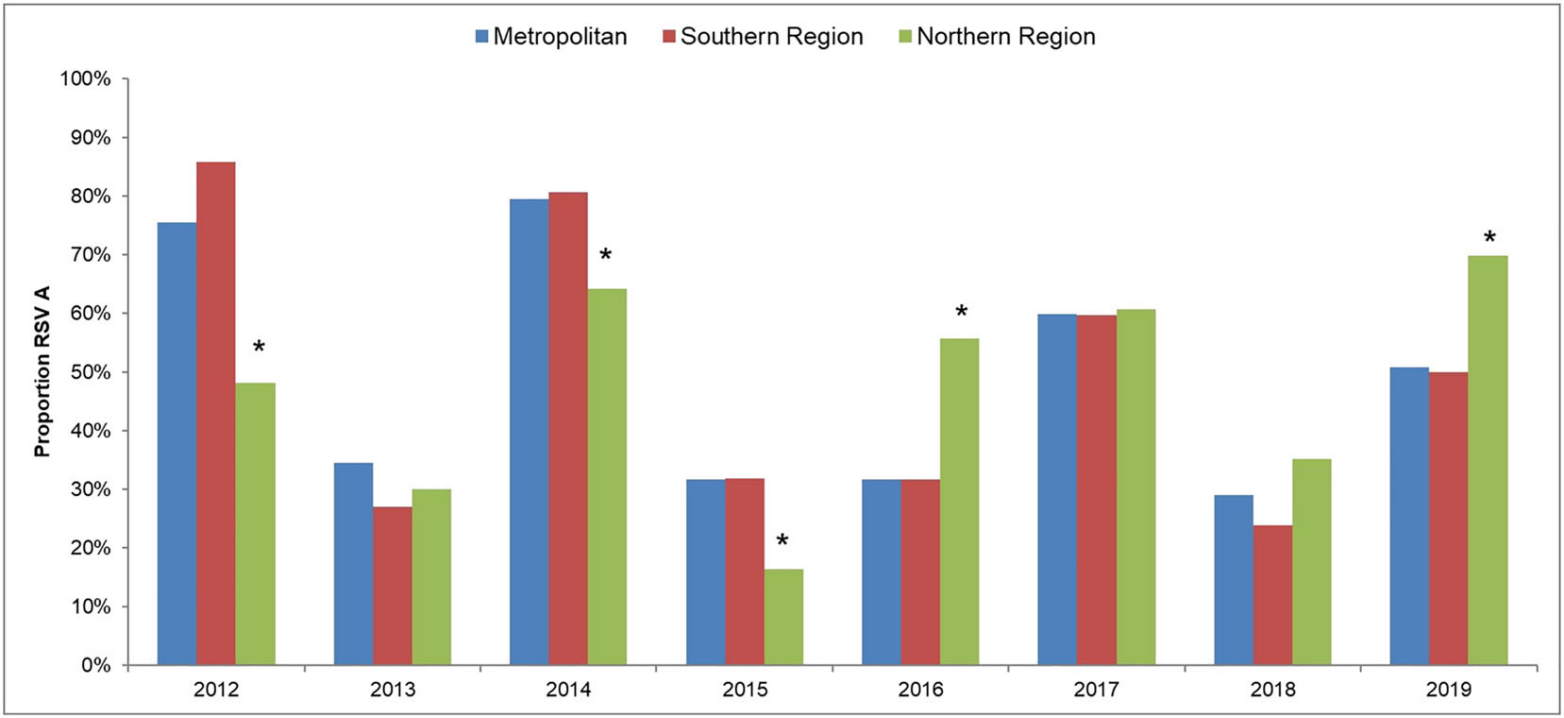
Proportion of RSV A cases compared with RSV B in each region by year. * denotes proportion was significantly different (*p* < 0.05) to the other regions for that year.

RSV subtype data was partially available for 2012–2016 and 2019 (between 32% and 65%) and almost complete for 2017–2018 (95%). RSV A was the dominant subtype in 2012, 2014 and 2017, while RSV B was dominant in 2013, 2015, 2016 and 2018. RSV A and B were co‐circulating in similar proportions in 2019. The proportions of RSV A to RSV B were similar for each year's Metropolitan and Southern regions. However, the proportion of RSV A to RSV B in the Northern region significantly differed from the Metropolitan region in 5 out of 8 years studied (Figure 4).

No difference was seen in RSV season timing when analysing RSV A and RSV B dominant seasons separately.

## 4 DISCUSSION

This report examined retrospective laboratory data to determine RSV detection rate and seasonality in Western Australia. There were 6.3 cases of RSV per 10,000 population in WA during the study period. The highest detection rate was observed in the Northern region, reaching a mean of 15 cases per 10,000, with a DRR of 2.7 (95% CI, 2.6–2.9) compared with the Metropolitan region. Test percentage positivity was 8.6% in WA, with the lowest positivity in the Northern region (8.1%). RSV seasons in the sub‐tropical to temperate Metropolitan and Southern regions occur annually, with a single peak and consistent timing and intensity. The Northern tropical region did not experience a distinct RSV season but had ongoing RSV activity throughout the year. Further, the proportion of RSV sub‐groups observed in the Northern region significantly differed from the Metropolitan region in over 60% of seasons.

The highest case rates were observed in the Northern region, more than 2.5 times higher than in the Metropolitan region. The local climate conditions and the absence of a defined season may have expanded the opportunity for viral transmission. However, case detection may have been boosted by other factors. The Northern region has a high Aboriginal population; therefore, proportion of the population from groups at risk of RSV‐related hospitalisation is significantly higher than in other regions in WA.^17^ Further, percentage positivity was lower in this region, suggesting that testing practices, in part, may account for some of this difference.

Between 2012 and 2019, a single annual RSV season was observed in the Metropolitan and Southern regions. This finding contrasts with an earlier report of RSV seasonality in WA, which reported a biennial seasonal pattern from 2000 to 2005.^18^ The shift to a consistent single peak could be attributed to the change in the local population. The Metropolitan region's population expanded rapidly over the last two decades, with increased absolute numbers and population density,^19^ strengthening chains of transmission. This single peak in RSV cases is consistent with previous reports from other temperate regions.^4^, ^5^, ^16^ However, the stability of season onset and peak in the Metropolitan region and, to a lesser extent, the Southern region appears to be uncommon, with other temperate regions experiencing more variability in their season timing.^4^, ^16^ Perth has minimal year‐to‐year climate variability, with July being the coldest, wettest month.^20^ This predictability, combined with an endemic northern reservoir, may contribute to stable seasonality.

The Northern region did not experience a distinct RSV season but had ongoing RSV activity throughout the year. Previous studies have shown RSV seasonality in tropical regions to be diverse,^4^, ^5^ with some tropical regions such as Brazil and Thailand experiencing distinct RSV seasons,^4^, ^21^ while others such as Cote D'Ivoire had no observed seasonality.^4^ The Northern Territory of Australia, a tropical region adjacent to the Northern region of WA, has reported a distinct RSV season in their rainy season.^22^ This phenomenon of differing RSV seasonality in nearby geographical locations of similar climates has also been reported previously in Kenya.^23^ However, parts of the Northern region and the Top End of the State of Northern Territory experience monsoon climates, the average rainfall during the wet season of the Northern Territory is more than twice that of the Northern parts of WA,^24^ which may contribute to the different RSV seasonality between these regions. It is also noted that the total number of cases in the Northern region in this study was low overall, which may preclude seasonality from being apparent.

WA's remarkable consistency in RSV seasonality was disrupted in 2020 with the emergence of SARS‐CoV‐2 and the implementation of travel restrictions, border closures and stay‐at‐home orders in WA. The Perth metropolitan region experienced a near absence of RSV during the expected winter RSV season,^25^ followed by a late and intense peak of cases in the summer of 2020/2021.^7^ This region's observed consistent RSV seasonality further underlines the unusual RSV activity seen in 2020. If it occurs, the transition back to previous stable patterns will provide valuable information on factors influencing RSV seasonality.

The proportion of RSV A to RSV B in all years studied was similar in the Metropolitan and Southern regions. In five seasons, the proportion was significantly different in the northern regions. This finding suggests that the Northern region may have different circulating RSV strains. Previous genomic studies have shown that RSV strains from WA are dissimilar to those from the east coast of Australia.^26^, ^27^ However, analysis has not been performed to compare regions within WA.

The strength of this study is that it utilised 8 years of laboratory RSV tests and case data to look for seasonal patterns. During this time, the criteria for sample collection and laboratory testing practices remained relatively stable. Together, these factors allowed for a long‐term picture of RSV activity to be established for WA. In addition to this, the large geographical area that was captured allowed for RSV activity to be compared across different climatic regions and areas of different population densities.

This study had several limitations. While subtyping data were available for a proportion of the RSV cases, it was not complete, and the proportions of available data varied from year to year. A complete dataset of subtyping data would have allowed for a more in‐depth analysis of the effects subtype may have had on seasonality. Testing practices may differ between regions and may have changed during this study, influencing findings. PathWest is the majority pathology provider for the WA regions, capturing both community and hospital attending patients; whereas, in the Perth metropolitan area, PathWest mainly tests hospital attending patients, with community patients being tested by private pathology providers. This difference in test population between the regions may have influenced the results with mild cases being undercounted in the metropolitan region. Population data for 2016 was used to calculate detection rate per 10,000 population. It is possible that the population of these regions varied from this midpoint during the study period, which may have had an impact on the results; however, it is expected that this impact is small.

## 5 CONCLUSION

Prior to the introduction of SARS‐CoV‐2 non‐pharmaceutical interventions, RSV seasonality in WA was consistent in its timing and intensity for the Metropolitan and Southern regions. No defined seasonality was seen in the Northern region. The differing patterns between regions in WA and the disruption of these patterns by the SARS‐CoV‐2 pandemic highlight the importance of ongoing RSV surveillance to improve our understanding of respiratory virus seasonality.

## AUTHOR CONTRIBUTIONS

Cara A. Minney‐Smith: Writing—original draft (lead); conceptualisation; data curation; investigation; methodology; visualisation; formal analysis; project administration. David A. Foley: Writing—original draft; visualisation; investigation; methodology; and formal analysis. Chisha T. Sikazwe: Data curation; methodology; writing—review and editing. Avram Levy: Conceptualisation; data curation; investigation; methodology; supervision; writing—review and editing. David W. Smith: Conceptualisation; methodology; supervision; writing—review and editing.

## CONFLICT OF INTEREST

David W. Smith is Director of the Asia‐Pacific Alliance for the Control of Influenza, an independent not‐for‐profit organisation that receives funding from vaccine manufacturers. The position is unpaid and he receives only reimbursement of expenses.

### PEER REVIEW

The peer review history for this article is available at https://publons.com/publon/10.1111/irv.13117.

## Supporting information


**Table S1:** RSV cases and number of tests by region per year. Population data is based on 2016 census data.

## Data Availability

The data that support the findings of this study are available on request from the corresponding author. The data are not publicly available due to privacy or ethical restrictions.
