# Supplementary material for: The seasonality of respiratory syncytial virus in Western Australia prior to implementation of SARS‐CoV‐2 non‐pharmaceutical interventions
Source: Influenza Other Respir Viruses. 2023 Mar 9;17(3):e13117. doi: 10.1111/irv.13117 (PMC10035409; doi:10.1111/irv.13117)
Supplement: Supplementary file 1 — Table S1: RSV cases and number of tests by region per year. Population data is based on 2016 census data. [file IRV-17-e13117-s001.docx]

Supplemental Data

|  | Perth  (Population 1,943,858) | | Southern Region  (Population 430,247) | | Northern Region  (Population 93,920) | |
| --- | --- | --- | --- | --- | --- | --- |
|  | *Tests* | *Cases* | *Tests* | *Cases* | *Tests* | *Cases* |
| 2012 | 11666 | 1032 | 3051 | 345 | 1453 | 143 |
| 2013 | 11474 | 1029 | 2790 | 275 | 1109 | 105 |
| 2014 | 13572 | 1093 | 3807 | 292 | 1848 | 141 |
| 2015 | 15378 | 1404 | 4293 | 348 | 1590 | 143 |
| 2016 | 14501 | 1177 | 4045 | 326 | 1908 | 157 |
| 2017 | 9120 | 830 | 3659 | 322 | 1775 | 119 |
| 2018 | 8941 | 869 | 3902 | 341 | 1539 | 125 |
| 2019 | 13986 | 1081 | 6409 | 532 | 2774 | 195 |
| TOTAL | 98638 | 8515 | 31956 | 2781 | 13996 | 1128 |

Supplemental Table 1: RSV cases and number of tests by region per year. Population data is based on 2016 census data.
